# Supplementary material for: Estimating microbial population data from optical density
Source: PLoS One. 2022 Oct 13;17(10):e0276040. doi: 10.1371/journal.pone.0276040 (PMC9562214; doi:10.1371/journal.pone.0276040)
Supplement: S1 File — PDF file that contains step by step instructions on how to perform a plate reader calibration published on protocols.io. (PDF) [file pone.0276040.s001.pdf]

Estimating microbial population data from optical density V.1

Portia M Mira[1], Pamela Yeh[1], Barry G. Hall[2]

[1]Ecology and Evolutionary Biology, UCLA; [2]Bellingham Research Institute

Version 1

Oct 01, 2022

1 Works for me Share

dx.doi.org/10.17504/protocols.io.8epv5j6wjl1b/v1

Portia M Mira
Ecology and Evolutionary Biology, UCLA

ABSTRACT

The spectrophotometer has been used for decades to measure the density of bacterial populations as the turbidity expressed as optical density – OD. However, the OD alone is an unreliable metric and is only proportionately accurate to cell titers to about an OD of 0.1. The relationship between OD and cell titer depends on the configuration of the spectrophotometer, the length of the light path through the culture, the size of the bacterial cells, and the cell culture density. We demonstrate the importance of plate reader calibration to identify the exact relationship between OD and cells/mL. We use four bacterial genera and two sizes of micro-titer plates (96-well and 384-well) to show that the cell/ml per unit OD depends heavily on the bacterial cell size and plate size. We applied our calibration curve to real growth curve data and conclude the cells/mL – rather than OD – is a metric that can be used to directly compare results across experiments, labs, instruments, and species.

DOI

dx.doi.org/10.17504/protocols.io.8epv5j6wjl1b/v1

PROTOCOL CITATION

Portia M Mira, Pamela Yeh, Barry G. Hall 2022. Estimating microbial population data from optical density. **protocols.io**
https://dx.doi.org/10.17504/protocols.io.8epv5j6wjl1b/v1
Version created by Portia M Mira

LICENSE

This is an open access protocol distributed under the terms of the Creative Commons Attribution License, which permits unrestricted use, distribution, and reproduction in any medium, provided the original author and source are credited

CREATED

Oct 01, 2022

Citation: Portia M Mira, Pamela Yeh, Barry G. Hall Estimating microbial population data from optical density https://dx.doi.org/10.17504/protocols.io.8epv5j6wjl1b/v1

This is an open access protocol distributed under the terms of the **Creative Commons Attribution License** (https://creativecommons.org/licenses/by/4.0/), which permits unrestricted use, distribution, and reproduction in any medium,

LAST MODIFIED

Oct 01, 2022

PROTOCOL INTEGER ID

70725

1

Preparation

When calibrating a microtiter plate reader for microbial growth experiments, it is essential to begin with
a culture in which almost all the particles (cells) can form colonies, i.e., are colony-forming units or
CFU. To achieve that, a mid-log phase culture is required - one in which the culture has not begun to
enter stationary phase.

There are two easy ways to prepare overnight cultures that are highly reproducible and contain almost
exclusively viable cells. Both methods involve limiting cell growth before stationary phase has begun.

1.1 Method 1: Using rich broth media to obtain oxygen-limited cultures

Inoculate a colony into 10 ml of your favorite broth medium in a 15 ml plug-seal centrifuge tube, then
tighten down the plug-seal cap and allow the culture to stand overnight at the preferred growth
temperature without shaking or other method of aeration.

The details are important. We do not want the culture to be aerated because we want to maintain the
culture at exponential or early stationary phase. Using the 10 ml of medium in a 15 ml tube allows only
5 ml of oxygen-containing head space. The plug-seal cap ensures that no additional oxygen is available
to the growing culture.

1.2 A mineral salts medium, such as M9 medium, containing a limiting concentration of a carbon source
such as glucose (0.02% w/v) works well for many organisms. Inoculate a suitable volume of carbonlimited
medium with a single colony and shake well overnight at the optimum growth temperature.

Citation: Portia M Mira, Pamela Yeh, Barry G. Hall Estimating microbial population data from optical density
https://dx.doi.org/10.17504/protocols.io.8epv5j6wjl1b/v1

This is an open access protocol distributed under the terms of the **Creative Commons Attribution License**
(https://creativecommons.org/licenses/by/4.0/), which permits unrestricted use, distribution, and reproduction in any medium,

A couple of days before doing the calibration, grow either an oxygen limited or nutrient limited culture.
Measure the OD of that culture in 6 wells of a microtiter plate and the OD of the uninoculated medium
in another 6 wells (the blanks). Subtract the mean OD of the blank wells from the mean OD of the wells
containing the culture. That OD is the corrected OD of the nutrient or oxygen limited culture. Use
either a 96-well plate or a 384-well plate containing the volume you typically use in that plate. For this
purpose, it doesn't matter which size plate you use.

The OD units are OD times volume in ml. For example, a 10 ml culture with a corrected OD of 0.3
contains 3 OD units. The purpose of using OD units is to let you determine the volume of limited culture
you will need for the real experiment.

2 Calibration
Grow a sufficient volume of limited culture to provide at least 10 OD units. For oxygen limited cultures
this may require several 10 ml cultures. If so, combine the cultures. Check the OD of the combined
culture to see that there are about 10 OD units.

2.1 Spin down the culture(s), pour off the supernatant, and resuspend the pellet in 4.0 ml (or more) of M9
buffer with no nutrients. This volume will depend on how much is needed for the calibration.

(We found that 4.0 ml was sufficient for both 96-well and 384-well calibrations.)

2.2 Vortex the resuspended cells to
mix well. This is the zero tube which should have an expected 10 OD units.

3 Serial Dilutions for OD Measurements

Prepare a series of tubes numbered 1-10, each containing 2.0 ml of M9 buffer.

3.1 Serially transfer 2.0 ml from tube zero to tube 1 and vortex to mix well.
Accurate dilutions require bothaccurate pipetting and thorough mixing at each step.

Citation: Portia M Mira, Pamela Yeh, Barry G. Hall Estimating microbial population data from optical density https://dx.doi.org/10.17504/protocols.io.8epv5j6wjl1b/v1

This is an open access protocol distributed under the terms of the **Creative Commons Attribution License** (https://creativecommons.org/licenses/by/4.0/), which permits unrestricted use, distribution, and reproduction in any medium,

3.2 Continue two-fold serial dilutions, transferring and mixing 2 ml at each step.

3.3 Fill the wells of a 96 well plate and/or a 384 well plate according to the plate layouts below.

We use 200µl per well for 96 well plates and 80 µl per well for 384 well plates, but you can use whatever volumes
you choose providing that you use the same volumes for your growth experiments.

3.4 96 well layout (The layouts provide 6 wells at each dilution)

A	B	C
	Wells 1-6	Wells 7-12
Row A	Tube 0	Tube 1
Row B	Tube 2	Tube 3
Row C	Tube 4	Tube 5
Row D	Tube 6	Tube 7
Row E	Tube 8	Tube 9
Row F	Tube 10	Buffer Blank
Row G	Empty	Empty
Row H	Empty	Empty

If tube zero was at OD = 2.5, then tube 10 will be at about OD = 0.002. In our hands the limit of reliable
OD reading is about 0.005, so the serial dilutions will span the useful range of OD.

A	B	C	D	E
	Wells 1-6	Wells 7-12	Wells 13-18	Wells 19-24
Row A	Tube 0	Tube	Tube 2	Tube 3
Row B	Tube 4	Tube 5	Tube 6	Tube 7
Row C	Tube 8	Tube 9	Tube 10	Buffer Blank

Of course, you can use whatever layout you prefer, but these layouts will allow you to use the
accompanying “Calibration Calculator” Excel spreadsheet for your calculations. This sheet can be located in the Supplemental Information files in the Plos Publication.

Citation: Portia M Mira, Pamela Yeh, Barry G. Hall Estimating microbial population data from optical density
https://dx.doi.org/10.17504/protocols.io.8epv5j6wjl1b/v1

This is an open access protocol distributed under the terms of the **Creative Commons Attribution License** (https://creativecommons.org/licenses/by/4.0/), which permits unrestricted use, distribution, and reproduction in any medium,

3.5 Read each plate in your microtiter plate reader

Read each plate at 5-minute intervals for 30 minutes, shaking as usual between readings. This allows the
plate reader to settle down.

3.6 Serial dilutions and plating for colony counts

From tube zero, prepare 10^4, 10^5, 10^6 and 10^7 - fold dilutions in M9 buffer so that you have at least 1.0
ml of each dilution. Again, pipette carefully and mix well at each dilution. Plate 100µl per plate onto 5
broth plate for each of the 10^4, 10^5, 10^6 and 10^7 - fold dilutions. (*Note- these dilutions will depend on your organism and may change slightly). Incubate the plates at the appropriate temperature until colonies have grown.

4 Analyze the Data

4.1 Colony Counts

Count the colonies on whichever dilution has the closest to an average of about 100 colonies per plate.
The viable cells per ml in the zero dilution is 10 x dilution x mean colonies per plate.

4.2 OD Readings

96 well plate, sheet 1

From the plate reader output file copy the 30-minute OD readings into a copy of the Calibration
Calculator excel file, cells D14 through I25.

Into cell A10 enter the dilution that gave closest to 100 colonies per plate. Into cell B10 enter the mean
number of colonies per plate for that dilution. Cell C10 will show the cells/ml in the zero tube.

Cells B14 - B24 will show the cells/ml in tubes 0 through 10.

J14-J25 will show the mean ODs in tube 1-10 and the buffer blank.

K14-K24 will show the mean corrected OD, i.e., mean OD - mean OD of the

Citation: Portia M Mira, Pamela Yeh, Barry G. Hall Estimating microbial population data from optical density https://dx.doi.org/10.17504/protocols.io.8epv5j6wjl1b/v1

This is an open access protocol distributed under the terms of the **Creative Commons Attribution License** (https://creativecommons.org/licenses/by/4.0/), which permits unrestricted use, distribution, and reproduction in any medium,

buffer blank.

4.3 384 well plate, sheet 2

From the plate reader output file copy the 30-minute OD readings into a copy of the Calibration
calculator excel file, cells D14 through I25.

Into cell A10 enter the dilution that gave closest to 100 colonies per plate. Into cell B10 enter the mean
number of colonies per plate for that dilution. Cell C10 will show the cells/ml in the zero tube.

Cells B14 - B24 will show the cells/ml in tubes 0 through 10.

J14-J25 will show the mean ODs in tube 1-10 and the buffer blank.

K14-K24 will show the mean corrected OD, i.e., mean OD - mean OD of the buffer blank.

5 Graph Mean Corrected OD vs Cells/ml and Fit Curve

You will need a graphing program that has curve-fitting functions.
We use DataGraph for MacOS (https://www.visualdatatools.com/DataGraph/) or KaleidaGraph for both Mac and Windows (https://www.synergy.com/). Other programs include GraphPad Prism (Mac and Windows) (https://www.graphpad.com/scientific-software/prism/) and MagicPlot (Mac, Windows and Unix)
(https://magicplot.com/).

Into your graphing program paste the Mean Corrected OD column down through values ≥0.005 and the
corresponding Cells/ml. Plot corrected OD (X-axis) vs Cells/ml (Y-axis) and fit a polynomial of degree
4 to those point. This will generate the equation that predicts cells/ml from OD.

The equation will be of the form cells/ml = (A x OD4) + (B x OD3) + (C x OD2) + (D x OD) + E, but will probably be displayed as AX4 _ BX3 + CX2 + DX + E. Some programs display the equation as
E + DX +CX2 + BX3 + AX4. However, it is displayed, in your favorite text editor create a .cal file that lists the coefficients, one per line, in the order A, B, C, D, E and be sure that the last line is blank.

We name the file based on the species and the number of wells in the plate; e.g. Eco384.cal for E.
coli in a 384 well plate.

Citation: Portia M Mira, Pamela Yeh, Barry G. Hall Estimating microbial population data from optical density
https://dx.doi.org/10.17504/protocols.io.8epv5j6wjl1b/v1

This is an open access protocol distributed under the terms of the **Creative Commons Attribution License**
(https://creativecommons.org/licenses/by/4.0/), which permits unrestricted use, distribution, and reproduction in any medium,

6 Example to illustrate using the Calibration Calculator

The file Calibration_384well_Ecoli.xlsx in the Calibration Tools folder is the output of an E. coli calibration of a serial dilution Tube 0 in tubes 1-15, and a buffer blank. The ODs from the 30-minute reading were copied into columns D-I, rows 14-29 of a Calibration Calculator file. The buffer blanks, cells E1-E6, were copied into cells D31-I31. The colony counts from a 106 dilution in which 0.1 ml samples were plated onto 5 LB plates had an average of 266 colonies per plate. The dilution and colony counts were entered in cells A10 and B10 respectively of the Calibration Calculator. The values of the cells/ml were automatically calculated in cells B14-B29. The ODs corrected for the blank were automatically calculated in columns J-O in rows 14-29, and the mean corrected ODs were automatically calculated in column P, rows 14-29.

Experience has shown that ODs < 0.005 are unreliable, so the mean corrected ODs in rows 14-24 were plotted vs the cells/ml in those same rows.

The polynomial order 4 fit of cells/ml (Y-axis) vs Mean corrected OD (X-axis) gave the equation cells/ml = 513817 + 1.002e9*x + 1.342e9*x^2 + (-2.301e9)*x^3 + 1.552e9*x^4 with R2 = 1.0

The resulting .cal file, Ecoli384.cal looks like this:

```
1.552e9
-2.301e9
1.342e9
1.002e9
513817
```


Citation: Portia M Mira, Pamela Yeh, Barry G. Hall Estimating microbial population data from optical density
https://dx.doi.org/10.17504/protocols.io.8epv5j6wjl1b/v1

This is an open access protocol distributed under the terms of the **Creative Commons Attribution License** (https://creativecommons.org/licenses/by/4.0/), which permits unrestricted use, distribution, and reproduction in any medium,
